# Identification of Land-Cover Characteristics Using MODIS Time Series Data: An Application in the Yangtze River Estuary

**DOI:** 10.1371/journal.pone.0070079

**Published:** 2013-07-24

**Authors:** Mo-Qian Zhang, Hai-Qiang Guo, Xiao Xie, Ting-Ting Zhang, Zu-Tao Ouyang, Bin Zhao

**Affiliations:** Coastal Ecosystems Research Station of the Yangtze River Estuary, Ministry of Education Key Laboratory for Biodiversity Science and Ecological Engineering, Institute of Biodiversity Science, Fudan University, Shanghai, P.R. China; The Ohio State University, United States of America

## Abstract

Land-cover characteristics have been considered in many ecological studies. Methods to identify these characteristics by using remotely sensed time series data have previously been proposed. However, these methods often have a mathematical basis, and more effort is required to better illustrate the ecological meanings of land-cover characteristics. In this study, a method for identifying these characteristics was proposed from the ecological perspective of sustained vegetation growth trend. Improvement was also made in parameter extraction, inspired by a method used for determining the hyperspectral red edge position. Five land-cover types were chosen to represent various ecosystem growth patterns and MODIS time series data were adopted for analysis. The results show that the extracted parameters can reflect ecosystem growth patterns and portray ecosystem traits such as vegetation growth strategy and ecosystem growth situations.

## Introduction

Land-cover characteristics and their dynamics have captured much attention in the field of ecology, since land-cover exerts a huge influence over ecosystem biodiversity, water budget [1], energy flow [2], and carbon cycling [3]. Remotely sensed time series data provide an opportunity to identify land-cover characteristics at the temporal scale, which often reflect the features of ecosystem growth patterns. Ecosystem growth patterns can be categorized into four types (adapted from [4]): (i) undisturbed ecosystems; (ii) ecosystems that have suffered coverage damage that either lasted the whole growing season or followed by vegetation restoration in the growing season; (iii) ecosystems that have suffered a phenology change that is expressed as either a shift in the growing season or a shortened growing season; and (iv) ecosystems that underwent changes in both coverage and phenology. However, it is challenging to extract desired land-cover characteristics while remaining independent of inter-annual and inter-class variations [1]. Therefore, proper land-cover characteristic identification methods are needed.

Methods that take into account the temporal features of time series data to identify land-cover characteristics have been developed in recent decades; such methods can be roughly classified into two types. The first type is based on signals observed at different temporal scales: vegetation information is often present at seasonal and inter-annual scales, while noise typically has a higher frequency. By decomposing data into different temporal frequencies, noises can be excluded and parameters can be obtained to reflect long-term trends or seasonal patterns. Research based on this kind of method includes land-cover classification [5] and long-term vegetation dynamic study [6]. However, the ecological meaning of parameters obtained by this kind of method is often limited, and the relations between parameters and land-cover dynamics need further investigation. The second type of methods is based on land surface phenological stages. The phenological stages recognized by time series data include: (i) constant low/no leaf period in winter when the vegetation is dormant, (ii) rapid vegetation growth period in spring, (iii) a period with relatively stable high aboveground biomass in summer, and (iv) rapid senescence period in autumn [7]. Research based on such methods can provide more detailed ecological information (Table 1) that can be applied to study land surface phenology [8], vegetation response to changing climate [9], zoology [10], and so on.

**Table 1:** Summary of vegetation metrics used in time series analysis.

Vegetation metric	Interpretation	References
Greenup	Time represents the start of growing season when plant grows and photosynthesis begins	[10], [21], [22]
Maturity	Time when green leaf area stabilizes with high photosynthesis activity	[22]
Senescence	Time when plant begins senescence either expressed by green biomass decrease orreduced photosynthesis	[22]
Dormancy	Time represents the end of growing season when photosynthesis reaches its minimum andplants become dominant	[10], [21], [22]
Length of growing season	Time span between greenup and dormancy which represents the duration of photosyntheticactivity	[10], [21]
Maximum VI	Highest VIs level in growing season	[21]
Timing of maximum VI	Time when VIs reaches its maximum	[10], [21]
Seasonal amplitude	VIs value difference between vegetation dormancy and have the highest aboveground biomass	[10], [21]
Annual integration	Sum of VIs values in growing season	[21]
Greenup rate	Growth rate during the period between greenup and mature	[10], [21]
Senescence rate	Senescence rate during the period between senescence and dormancy	[10], [21]

Though methods based on phenological stages have been widely used in ecological studies, phenological stages are often detected based on mathematical criteria such as choosing a certain threshold or detecting curve changes [8], [11]. However, it is difficult to choose a mathematically ideal technique [11], and different analysis methods sometimes provide conflicting results on the same research topic (such as the long-term greenup trend in North America [8]). In this study, we propose a method to identify land-cover characteristics from the ecological perspective of sustained vegetation growth. During the analysis, phenological growth stages were first identified based on sustained vegetation growth trends, and parameters designed to reflect land-cover characteristics were extracted accordingly. Improvement was also made in parameter extraction, which was inspired by a technique used for extracting the hyperspectral red edge position.

## Materials and Methods

### Ethics Statement

As a field survey conducted for remote sensing research, we did not conduct any activities concern field samplings of soil, plants, or animals in the work. All lands where we conducted the survey are non-fenced public areas and accessed to everyone, thus we do not need to ask for any official permission.

### Site Description

**Figure 1:**
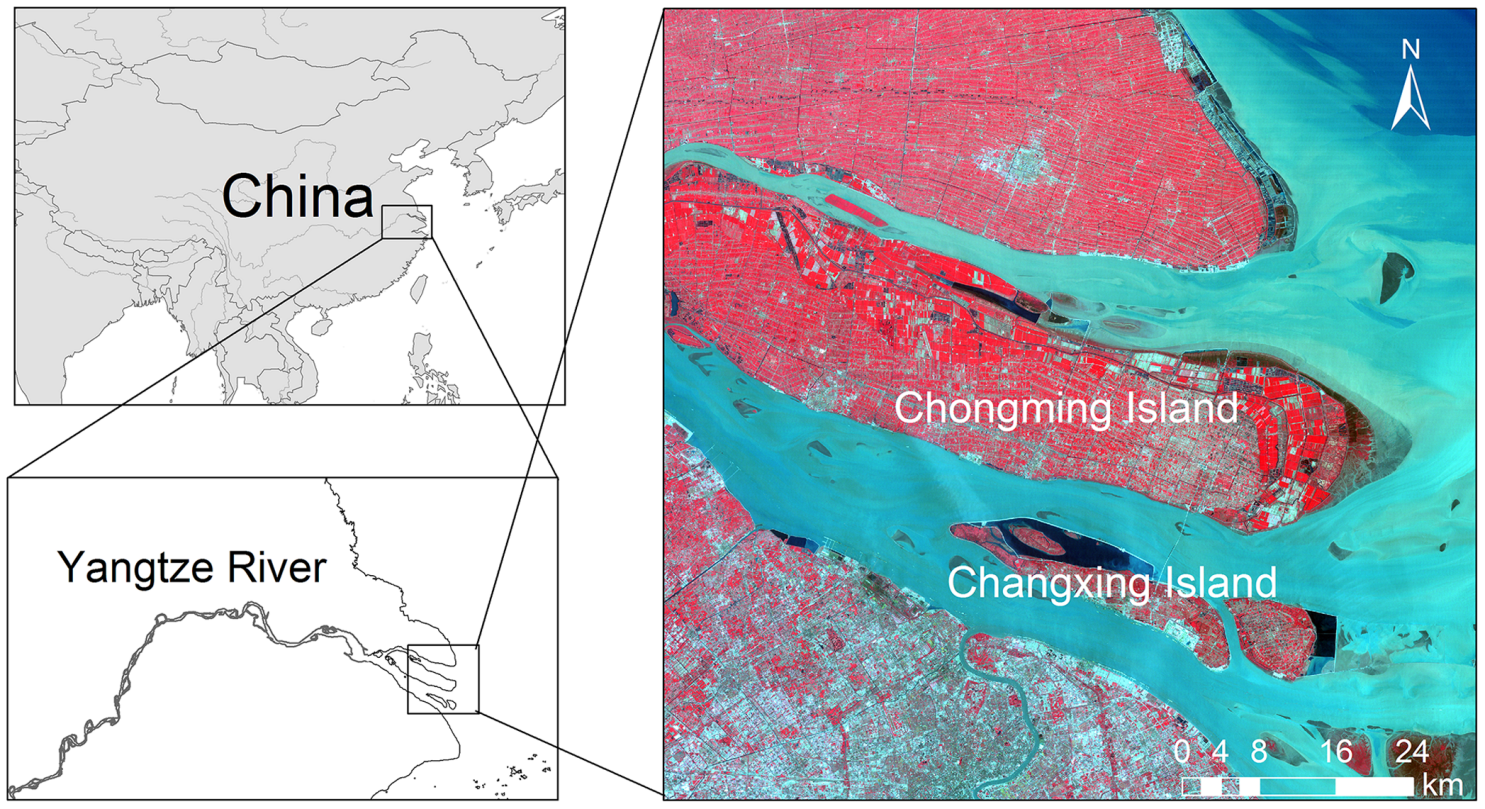
Location of the study area.

This study was conducted on the Chongming Island and the Changxing Island, two alluvial islands in the mouth of the Yangtze River, China (121°10′49″ –121°59′10″E, 31°17′4″ –31°54′20″N, Fig. 1). The area is subject to the northern subtropical monsoon climate, with an average annual temperature of 15.3°C and a total annual precipitation around of 1000 mm. Several large land reclamations have taken place since 1960s, the reclaimed areas are much larger than ordinary farmland and neighboring areas are often under the same land management schemes. Diverse land use and a relatively large reclamation area make the study area suitable for identifying land-cover characteristics with remote sensing data.

### Analysis Preparation

#### Remote sensing data

A 250 m 8-day composite surface reflectance data set (MOD09Q1) was used for this study. Satellite quality assurance (QA) data were obtained for further noise reduction, and selected data were derived from MOD09A1 because QA data from MOD09Q1 are insufficient to deliver the actual condition. Remote sensing data for the year 2009, when several field surveys were conducted, were used for analysis. All the remote sensing data used were downloaded from NASA (LP DAAC).

Vegetation indices (VIs) are specially designed indicators that reflect certain properties, such as vegetation coverage (*e.g*., NDVI, EVI & MSAVI) and land surface water content (*e.g*., LSWI). A two-band EVI (EVI2) was selected in this study for its superiority over the widely used NDVI [12]. EVI2 is calculated as follows:
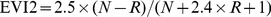
(1)where *N* and *R* are reflectance in the near-infrared (NIR) and red bands of MODIS data, respectively.

#### Field survey

In order to acquire the actual land-cover conditions in different seasons, we conducted three field surveys across the year of 2009. Before the first field survey, historical TM and airborne imageries were studied in the laboratory to identify the relatively homogenous regions for field surveys. During the field surveys, a portable Global Position System (GPS) was used to localize the target ground objects such as cultivated lands, fallow lands, orchards, and buildings. To aid this task, color maps of TM and airborne images were printed beforehand and taken with the investigators for field checks. The field notes were also made and taken to the laboratory for further analysis, such as location check, classification and accuracy assessment.

#### Land-cover selection

The studied land-cover types were chosen based on ecosystem growth patterns, and five land-cover types (urban, orchard, fallow, and two types of croplands) were chosen for further analysis (Table 2). Among them, urban, orchard, and fallow were used to represent ecosystems that experience a loss in coverage throughout the growing season; cropland-2 was used to represent ecosystems with short-term coverage loss; and cropland-1 was used to represent ecosystems under a growing season shift. Since in the study area no land-cover type showed the characteristics of ecosystems under a shortened growing season, this ecosystem growth pattern was not included in the present analysis. To better analyze land-cover characteristics, remote sensing pixels that represent only one land-cover type were used in the analysis.

**Table 2:** Descriptions of different land-cover types in study area.

Land-cover types	Description	Vegetation coverage	Disturbance pattern
**Urban**	Urban area	Low to medium	No
**Orchard**	Orange tree plantation area	Low to medium	No
**Fallow**	Farmland where no farming activities conducted, usuallycovered by natural herbaceous plants such as weed andcommon reed	Medium to high	No
**Cropland-1**	Single-cropping farmland with only rice planted from late Mayto October	High	Yes; Happened early in the year
**Cropland-2**	Double-cropping farmland with winter wheat planted from latelast November to early May and rice planted the same time as cropland1	High	Yes; Happened in the mid year

### Analysis Techniques

#### Noise reduction

**Figure 2:**
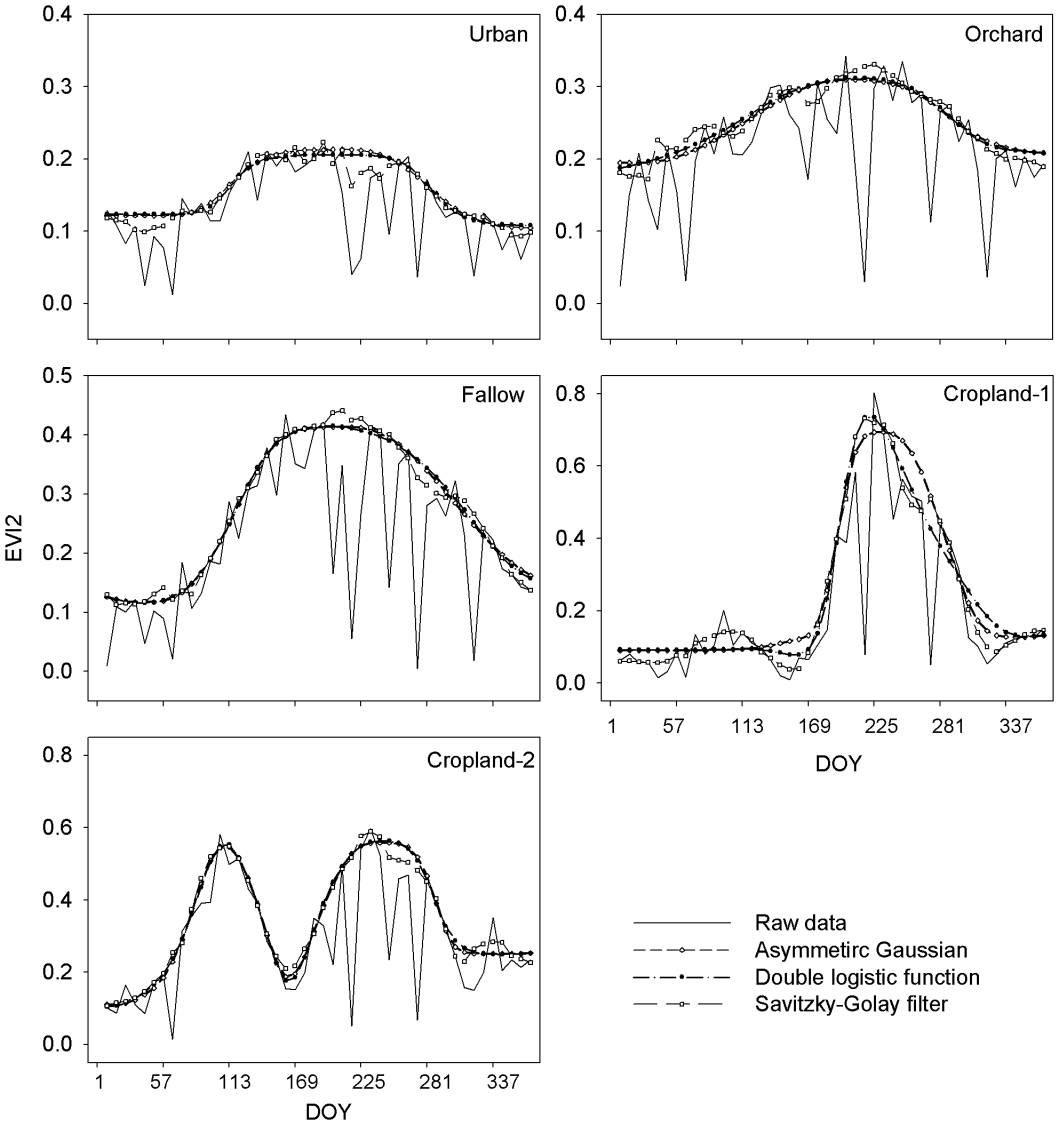
Time series EVI2 data of land cover types processed before and after noise reduction methods.

The asymmetric Gaussian method [13] and double logistic function [14] were chosen for noise reduction in this study, since their ability to maintain the integrity of signals is proven [15]. The Savitzky-Golay filter was also chosen since it could capture detailed variations in time series data and has shown good performance when applied in study related to China [16]. Noise reduction was achieved by using TIMESAT [13], [17], [18]. Ancillary weights of each data were set according to the QA data. Weights were set at high values for best-quality data (described as *clear* in QA data), at moderate values when data were acquired under less ideal conditions (*cloud shadow* or *mixed*), and at low values when data represent cloudy pixels. Fig. 2 shows the data of different land-cover types represented by EVI2 before and after noise reduction.

#### Phenological stages discrimination

Though rates of changes in vegetation coverage may vary, the vegetation growth trend inherited in each phenology stage (sustained increase/decrease, or consistency) remained constant for a certain time; therefore, we propose to discriminate phenological stages based on the sustained vegetation growth trend. The sustained trend was recognized by the following procedure: if the increment/decrement between neighboring data was larger than a certain numerical value (the theoretical increase/decrease threshold), we defined it as an increase or a decrease; and if the increment/decrement remained constant for some time (for instance more than one month), the period would be identified as showing a sustained increase/decrease trend. Time points (greenup, maturity, senescence, and dormancy; see Table 1) that separate these phenological stages were identified accordingly. Greenup and maturity were identified as the beginning and ending of the period when vegetation showed a sustained increase trend, respectively; the beginning and ending of the sustained decrease trend were termed as senescence and dormancy, respectively.

The theoretical increase/decrease threshold was calculated as:
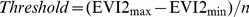
(2)where EVI2_max_ represents the maximum value of each time series data. Because the aboveground biomass of evergreen vegetation may vary in winter, when calculating the theoretical increase/decrease threshold, EVI2_min_ used the minimum value in the first/second half of the year, respectively. The variable *n* represents the period when vegetation biomass increases/decreases. The length of this period can be determined from long-term field observations. As the theoretical increase/decrease threshold is not supposed to give a quantitative value, the time period used can be longer than actual value. In this study, we simply assumed that the growing season spans the whole year, with vegetation biomass increase and decrease period accounting for half a year each. Further, the corresponding number of MODIS data was used to represent this period. If there were more than one sustained increase periods, the first period was used to identify greenup and maturity. Senescence and dormancy were identified in a similar way, except that the last sustained decrease period was used for the identification when more than one sustained decrease period existed.

#### Parameters extraction

**Figure 3:**
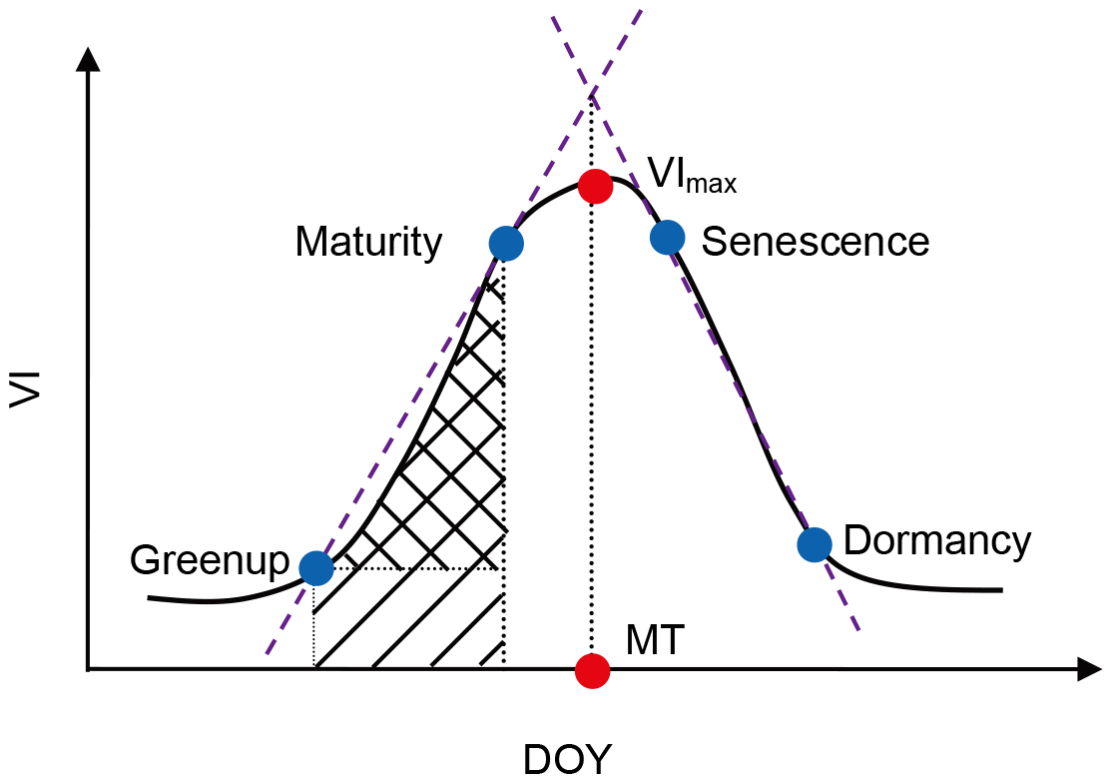
Diagram of parameter extraction from time series vegetation index (VI) data. Blue points represent time points separating different vegetation growth stages, while red points are parts of the extracted parameters.

The time at which aboveground biomass reaches its maximum (MT, a date) was first identified. MT was extracted by extrapolating two straight lines across the time points that discriminate phenological stages (Fig. 3). This process was inspired by a technique used in hyperspectral analysis, which stabilizes the red edge position when there are multiple peaks in the first derivative curve of hyperspectral data [19]. The EVI2 extracted on day MT was used to represent the maximum vegetation coverage (VI_max_, dimensionless). If MOD09Q1 data were missing for that day, VI_max_ was linearly interpolated between the previous and following data.

Two other parameters were further extracted to reflect the vegetation growth status. The average increase rate (AIR, dimensionless) between greenup and maturity was calculated to reflect how vegetation grows from the minimum vegetation coverage to a relatively stable status. The mean EVI2 between greenup and maturity (VI_gm_, dimensionless) focuses on the average status in the sustained growing period
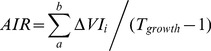
(3)

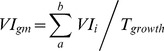
(4)where *a* and *b* represent greenup and maturity respectively; 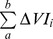 represents the accumulated increments of EVI2 in the sustained growing period (backslash region in Fig. 3); 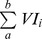 is the EVI2 accumulation in the same period (slash region in Fig. 3); *T_growth_* represents the time span between greenup and maturity (Fig. 3), and we used the number of MODIS data to represent this period.

Different ecosystem growth patterns can be expressed by parameter differentiations. Coverage differentiation would be most evident for ecosystems that have suffered vegetation coverage loss lasted the growing season, and the maximum vegetation coverage (hence the values of VI_max_) would then reduce accordingly. Since total vegetation coverage increase/decrease status is related to the maximum coverage, the values of AIR and VI_gm_ will also decrease. In ecosystems that are under short-term vegetation loss, VI_max_ will more or less represent the coverage during the period of vegetation damage and not the maximum coverage in the growing season; therefore, VI_max_ value will decrease. However, the changes in phenology or total vegetation coverage (and hence parameters of MT, AIR, and VI_gm_) depend on the severity and duration of the damage. Phenology differentiation is the most obvious characteristic of an ecosystem undergoing a growing season shift, and the value of MT would change accordingly. Ecosystems with shortened growing seasons exhibit slight shifts of phenology and accelerated vegetation coverage increase/decrease rates. All parameters would change in ecosystems that undergo both coverage and phenological changes.

#### Land-cover classification

**Figure 4:**
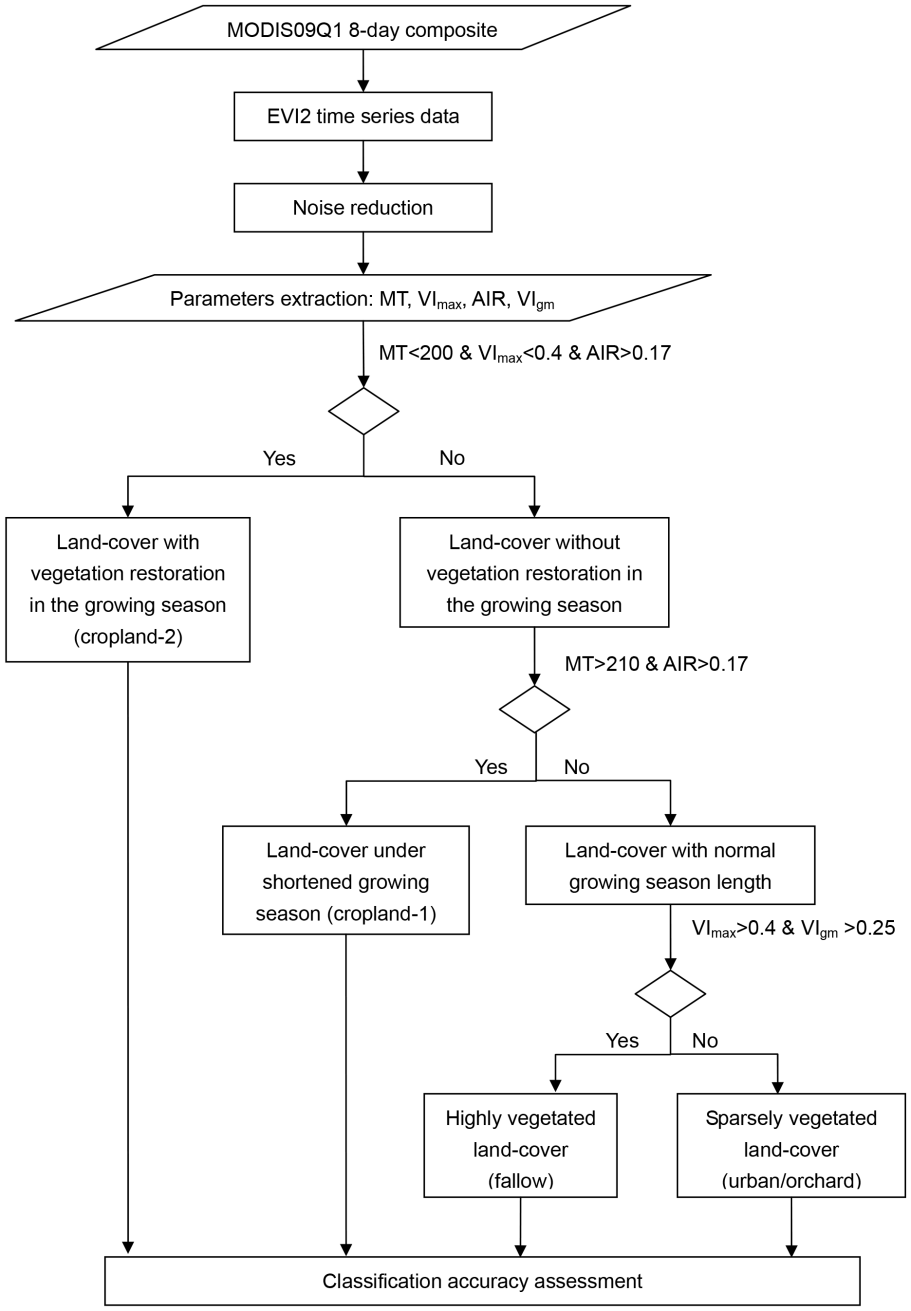
Workflow of hierarchical scheme for land-cover classification. All thresholds that been used in different noise reduction methods were same.

In order to test whether the extracted parameters could be used for actual land-cover change detection, a hierarchical classification scheme was adopted to classify the studied land-cover types (Fig. 4). The parameters used in each classification level were chosen based on the aim of the classification, with each parameter aimed to discriminate only one aspect of the land-cover (sparsely/densely planted, with/without phenological shift, or high/low growth rate). For example, in this study, both coverage and phenology in cropland-2 are distinct from other land-cover types, hence three parameters, MT, VI_max_, and AIR were chosen in the first classification level. Land-cover types were not sub-classified artificially from each other if no apparent differences in ecosystem features were detected.

The thresholds used for classification were set roughly according to predefined criteria rather than on the basis of training data, and hence all original data were used for validation. The thresholds were defined by the following criteria. The threshold of VI_max_/VI_gm_ was set as the arithmetic mean value of soil background and the highest/mean VI value of pixels with the highest vegetation coverage. MT, being representative of phenological information, would change in ecosystems under phenological changes. The threshold of AIR was set as the arithmetic mean value of the observed highest values and the lowest ones. All thresholds used in classification are the same for data processed by different noise reduction methods. Confusion matrixes were used to evaluate classification accuracies.

## Results

### Basic Characteristics of Land-cover Types

The extracted parameters can reflect the basic characteristics of different land-cover types (Table 3). MT value changes reflect changes in vegetation phenology. In cropland-1, MT values changes as the growing season has shifted, and these values are the largest among all land-cover types. In cropland-2, since human interference has actually altered vegetation phenology, the MT values have also shifted and are the smallest of all land-cover types. Urban, orchard, and fallow have intermediate MT values, which reflects the fact that the vegetation phenology has not changed here.

**Table 3:** The mean value and standard deviation (SD) of parameters extracted from time series vegetation index (VI) data with asymmetric Gaussian method (A), double logistic function (B), and Savitzky-Golay filter (C).

A	MT		VI_max_		AIR		VI_gm_	
	mean	SD	mean	SD	mean	SD	mean	SD
**Urban**	200	12.493	0.198	0.035	0.043	0.006	0.149	0.006
**Orchard**	205	31.842	0.370	0.047	0.059	0.021	0.308	0.041
**Fallow**	194	10.799	0.496	0.052	0.167	0.025	0.318	0.033
**Cropland-1**	228	6.799	0.618	0.082	0.250	0.049	0.391	0.035
**Cropland-2**	166	12.356	0.200	0.070	0.223	0.031	0.327	0.028
**B**	**MT**		**VI_max_**		**AIR**		**VI_gm_**	
	**mean**	**SD**	**mean**	**SD**	**mean**	**SD**	**mean**	**SD**
**Urban**	204	13.345	0.197	0.037	0.038	0.006	0.149	0.031
**Orchard**	216	26.139	0.372	0.047	0.053	0.019	0.310	0.043
**Fallow**	191	10.796	0.504	0.056	0.177	0.029	0.313	0.033
**Cropland-1**	229	10.878	0.625	0.085	0.252	0.067	0.390	0.040
**Cropland-2**	168	11.090	0.188	0.081	0.216	0.032	0.327	0.029
**C**	**MT**		**VI_max_**		**AIR**		**VI_gm_**	
	**mean**	**SD**	**mean**	**SD**	**mean**	**SD**	**mean**	**SD**
**Urban**	189	20.587	0.188	0.081	0.034	0.015	0.140	0.037
**Orchard**	185	40.105	0.350	0.052	0.063	0.025	0.290	0.055
**Fallow**	192	10.531	0.495	0.069	0.154	0.041	0.288	0.055
**Cropland-1**	228	9.770	0.628	0.092	0.285	0.077	0.349	0.047
**Cropland-2**	169	12.665	0.199	0.076	0.229	0.032	0.331	0.035

**Figure 5:**
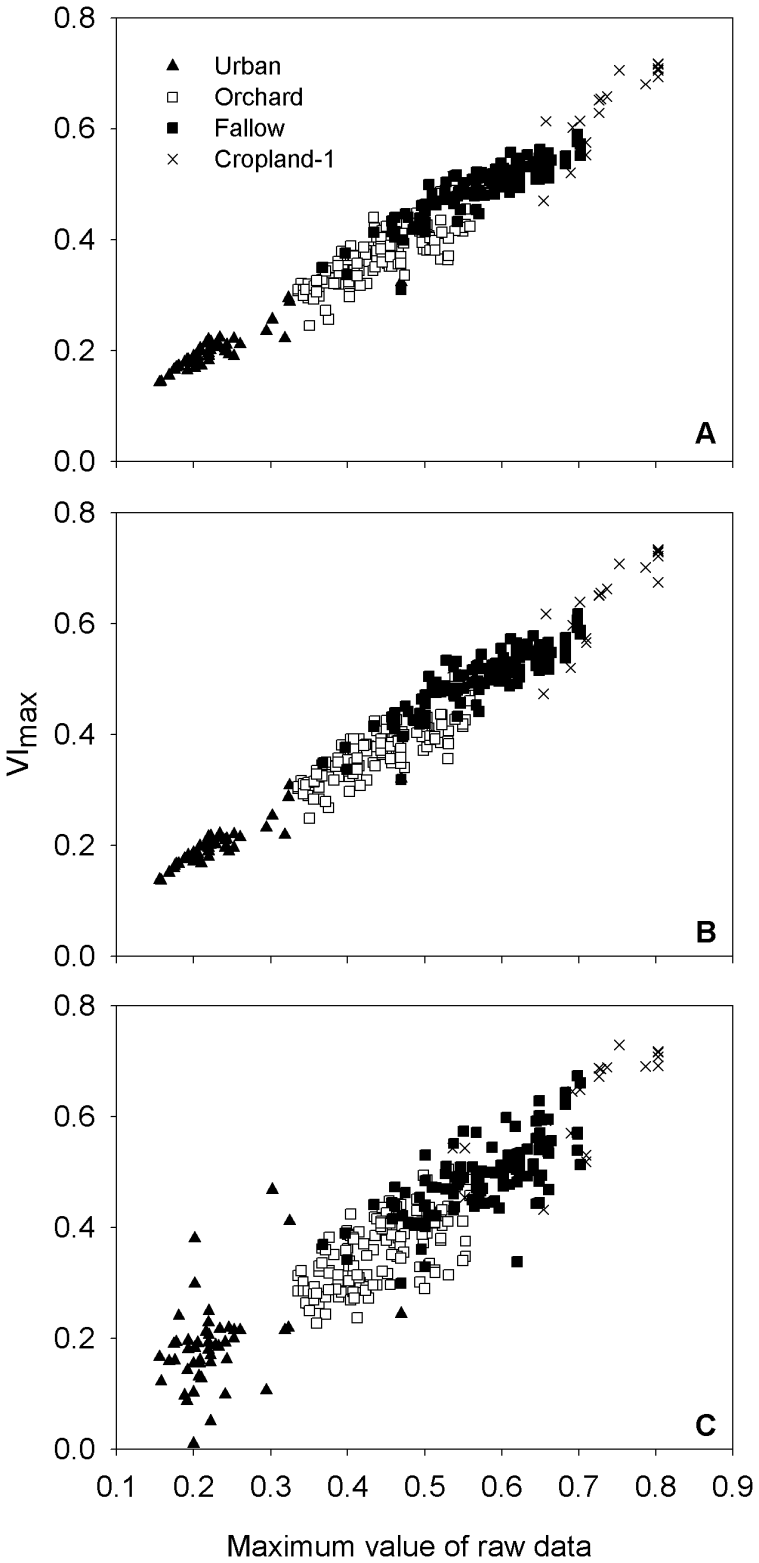
Results evaluation by comparing derived VI_max_ with corresponding maximum values of raw data. Frames showed results that obtained with asymmetric Gaussian method (A) (*R* ^2^ = 0.944), double logistic function (B) (*R* ^2^ = 0.947), and Savitzky-Golay filter (C) (*R* ^2^ = 0.837) respectively.

VI_max_ reflect the changes in ecosystem coverage. Since cropland-1 did not undergo vegetation coverage loss, this land-cover type has the highest VI_max_ values (Table 3). VI_max_ values of fallow, orchard, and urban decrease with reduced vegetation coverage. Because the tree density in orchard areas is not high, vegetation coverage of orchards is no larger than that of fallow areas (as indicated in Fig. 5); hence, it is understandable that the average VI_max_ values of orchard are lower than those of fallow. In cropland-2, as MT occurs during the time right after rice transplantation at when the land is barely covered, the values of VI_max_ are not high.

AIR and VI_gm_ are parameters that reflect vegetation growth status. On the whole, the change patterns of AIR and VI_gm_ values are similar to those of VI_max_, with cropland-1 having the highest values, followed by fallow and orchard, and urban areas having the smallest values. However, in cropland-2, as VI_max_ values do not reflect the maximum vegetation coverage in the growing season, AIR and VI_gm_ values do not follow the trend exhibited in VI_max_.

### MT Results Evaluation

An evaluation was performed on MT results to illustrate the variation in the values (Table 3), because although MT values are quite similar for data processed by different noise reduction methods in fallow, cropland-1, and cropland-2, the results of urban and orchard varied with methods and much lower MT values were obtained when using the Savitzky-Golay filter. As there is no readily available evaluation method, we chose an indirect means of assessment. VI_max_ is based on the position of MT, and a departure of MT from the time of the highest aboveground biomass would result in a decrease in the VI_max_ value. Thus, the VI_max_ value can serve as an indicator for MT evaluation, and comparisons of VI_max_ with the corresponding maximum values of raw data are shown in Fig. 5. The asymmetric Gaussian method and double logistic function provided satisfying results; however, the coefficient of the relationship (*R^2^*) is much lower when using the Savitzky-Golay filter, which indicates greater errors in data processed by this method. Therefore, the observed MT variations in Table 3 should be the result of unstable performance of the Savitzky-Golay filter when it is applied for areas with low vegetation coverage, as vegetation signals are weak and more sensitive to noise in such areas. Cropland-2 was excluded from this evaluation because its VI_max_ values do not reflect the maximum vegetation coverage in the growing season.

### Ecosystem Traits Detection

**Figure 6:**
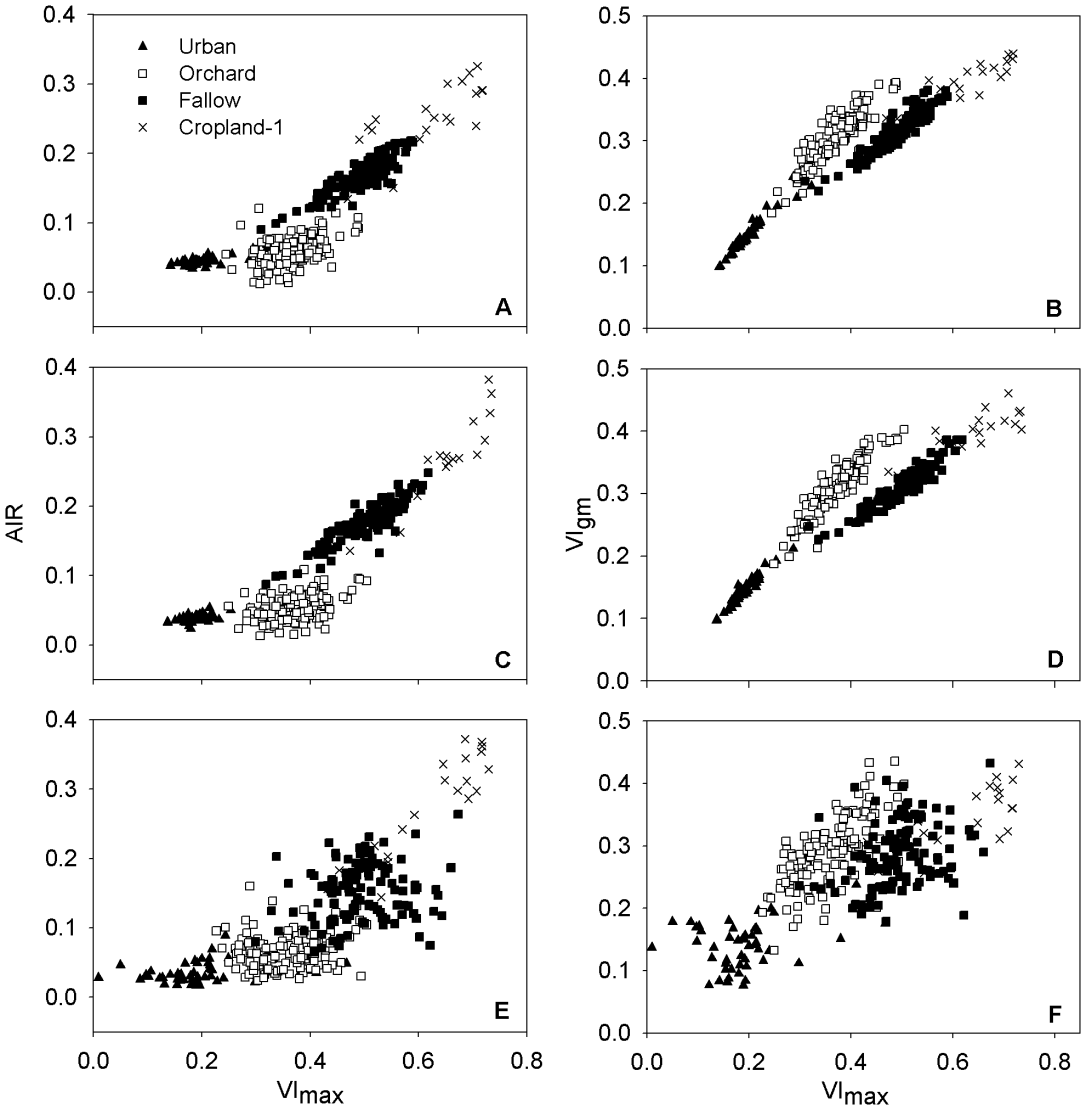
The cross-comparisons among parameters extracted. The left column represent the comparisons between AIR and VI_max_ obtained via asymmetric Gaussian method (A), double logistic function (C), and Savitzky-Golay filter (E), respectively. The right column represent the comparisons of VI_gm_ with VI_max_ by using asymmetric Gaussian method (B), double logistic function (D), and Savitzky-Golay filter (F).

Because the species composition varied among ecosystems, the vegetation growth condition expressed at ecosystem scale differed, and this trait is inherent in ecosystems. However, AIR and VI_gm_ cannot be used for direct detection of such differences, because the influence of coverage would hide them. By using VI_max_, the coverage differentiation can be partially minimized (Fig. 6), and vegetation growth traits can be conveyed through slope changes.

The differences between vegetation growth rates are shown in Fig. 6A, C, and E. In Fig. 6A and C, slopes of cropland-1 and fallow are larger than those of urban and orchard, indicating that under same coverage, cropland-1 and fallow grow faster. Although intra-class variations in data processed by Savitzky-Golay filter are larger than those in the data processed by the other two methods, a similar pattern could also be observed (Fig. 6E). Since orchard areas comprising woody plants and trees are the dominate urban vegetation in the study area, the slope differences seen in the figures indicate the differences between herbaceous vegetation and woody plants, as herbaceous plants grow faster that woody plants under suitable conditions.

The differences between ecosystem average growing conditions are shown in Fig. 6 B, D, and F. In these figures, the slopes of orchard are higher than those of cropland-1 and fallow, indicating that the average coverage of orchard is larger at ecosystem level. Since the woody plants in orchard are evergreen and herbaceous plants senescence every year, the aboveground biomasses are different when spring comes, therefore, the coverage of woody plants increased faster. Because some of the trees are deciduous in urban area, the slopes of urban are slightly lower than those of orchard. Cropland-2 was excluded in this part of analysis since its VI_max_ values do not reflect the maximum vegetation coverage in the growing season.

### Classification Accuracy Assessment

Confusion matrixes were used to evaluate classification accuracy, (Table 4) and were made for data processed by each different noise reduction method. All noise reduction methods achieve relatively high overall classification accuracy. The user’s accuracy of fallow is the lowest in all methods, and errors mainly arise from misclassification of urban/orchard. In our study, the chosen land-cover types are only based on actual land surface situations and we did not set any predefined coverage criterion for data selection. Therefore, the actual coverage of fallow, urban, and orchard can overlap (also indicated in Fig. 5), and hence misclassifications are acceptable.

**Table 4:** Land classification accuracy assessments of data processed by different smoothing methods (A) asymmetric Gaussian method, (B) double logistic function, and (C) Savitzky–Golay filter.

A	Reference data					
Classification	Urban/orchard	Fallow	Cropland-1	Cropland-2	Total	User’s accuracy (%)
**Urban/orchard**	144	5	0	29	178	80.90
**Fallow**	36	115	2	9	162	70.99
**Cropland-1**	0	2	19	0	21	90.48
**Cropland-2**	0	0	0	439	439	100
**Total**	180	122	21	477		
**Producer’s accuracy (%)**	80	94.26	90.48	92.03		
**Overall accuracy: 89.63%**		**Kappa: 0.825**				
**B**	**Reference data**					
**Classification**	**Urban/orchard**	**Fallow**	**Cropland-1**	**Cropland-2**	**Total**	**User’s accuracy (%)**
**Urban/orchard**	147	5	0	34	186	79.03
**Fallow**	33	117	2	16	168	69.64
**Cropland-1**	0	0	19	0	19	100
**Cropland-2**	0	0	0	427	427	100
**Total**	180	122	21	477		
**Producer’s accuracy (%)**	81.67	95.90	90.48	89.52		
**Overall accuracy: 88.75%**		**Kappa: 0.811**				
**C**	**Reference data**					
**Classification**	**Urban/orchard**	**Fallow**	**Cropland-1**	**Cropland-2**	**Total**	**User’s accuracy (%)**
**Urban/orchard**	144	29	0	18	191	75.39
**Fallow**	36	91	2	13	142	64.08
**Cropland-1**	0	1	19	0	20	95
**Cropland-2**	0	1	0	446	447	99.78
**Total**	180	122	21	477		
**Producer’s accuracy (%)**	80	74.59	90.48	93.50		
**Overall accuracy: 87.5%**		**Kappa: 0.786**				

Integers in tables represent the number of pixels that belongs to a certain classification condition.

## Discussion

### Phenological Stages Identification

Remote sensing phenological stages that used to extract land-cover characteristics are often identified by discriminating the time points that separate them. However, it is difficult to choose a mathematically ideal method [11]. Furthermore, as these points are timings that represent dynamic vegetation growth conditions, it is difficult to directly evaluate the results from field phenology observations, because the two kinds of data are not measured at the same spatial scale and often represent different ground phenological events [8]. Therefore, we turned to the sustained vegetation growth trend that phenological stages inherently exhibited, and time points were thus identified. In this identification process, as the theoretical increase/decrease threshold is not to give a precise quantitative value, the process can be flexible when applied to large scale analysis. Besides, this method can adjust itself according to the maximum and minimum values of the time series data of each pixel. Although time points were only used for later land-cover characteristic identification in this study, they can also be used in land surface phenology research.

### Land-cover Characteristics Identification

Parameters were extracted to reflect land-cover characteristics. In this study, the time of highest vegetation biomass (MT) was detected first, and the VI value that represented maximum vegetation coverage (VI_max_) was identified accordingly. Compared with commonly used methods [10], MT was extracted based on temporal features of time series data rather than by using a single maximum value, this makes it more resistant to variations caused by noise. In ecosystems where growth patterns change as a result of disturbances (either in coverage or phenology caused by events such as insect defoliation, windfall, and wildfire), the time points and therefore MT would change consequently; hence, MT can also be used as an indicator of disturbances. This is especially convenient if an irregular growing season was caused by such a disturbance. When MT is combined with VI_max_, subtle vegetation damages can be more evident. However, the time points of greenup and dormancy are sensitive to the start of spring and the end of autumn, which make these time points vulnerable to inter-annual meteorological variations. In order to obtain a more stable inter-annual result of MT, adjustments such as use of meteorology data or reference area are recommended.

AIR and VI_gm_ can portray ecosystem traits that represent how ecosystems grow. The trait difference between ecosystems with different vegetation composition is especially evident when coverage differences are minimized (Fig. 6). Though effort has been made to discriminate land-cover types that have different species composition by comparing growing season NDVI [20], this method can further explore the temporal features of ecosystems. Therefore, this method has potential for monitoring land-cover changes caused by species variation (such as species invasion and vegetation succession). Similar parameters extracted from vegetation biomass decrease period can also be used for detecting how vegetation senescence. This kind of information could help us to understand ecosystem changes in more detail, and help us to further explore ecosystem processes and functions, as well as the causes of the ecosystem changes.

### Land-cover Classification

As the extracted parameters incorporated both spectral and temporal features, land-cover characteristics can be better explored. Results of this study show that this kind of land-cover classification can achieve relatively satisfying results in practice. Classification schemes that include these parameters will facilitate land-cover mapping in complicated situations, such as in regions where the differences between land-cover types are subtle, or in areas with irregular growing seasons.

### The Performance of Noise Reduction Methods

Although an 8-day composition scheme is adopted in MODIS products, the presence of cloud remains a problem in retrieving land-cover characteristics in our study area. Therefore, the performance of noise reduction methods affects the ultimate results. Our results show that the asymmetric Gaussian method and double logistic function performed better than the Savitzky-Golay filter, and that some apparent discrepancies exist in the Savitzky-Golay filter. For example, in Fig. 5C some VI_max_ values in urban are obviously larger than the maximum values of raw data (such as 0.380 of VI_max_ corresponds to 0.201 of maximum raw data). This indicates larger errors in the noise reduced data, and indicates that the Savitzky-Golay filter is less robust in areas where vegetation is sparse and noises are frequent. It further confirms a conclusion obtained by [15] that the asymmetric Gaussian method and double logistic function can maintain the integrity of signals and that the Savitzky-Golay filter is sensitive to noise. Our results also give a direct illustration that the Savitzky-Golay filter is not suitable to deal with noise contaminated data at the seashore.

### Conclusion and Outlook

In this study, we tried to identify land-cover characteristics based on the consideration of sustained vegetation growth trends. During this process, an improvement was also made by simulating a method used for determining the hyperspectral red edge position. Our results show that this method can capture ecosystem growth patterns and more detailed ecosystem traits such as species growing strategy and ecosystem growth status. This method has a potential in land-cover dynamic studies related to vegetation coverage and composition changes (such as ecosystem damage evaluation, invasive species monitoring, and vegetation succession validation), and also in land surface phenology monitoring. When combined with auxiliary data, such as soil properties, or carbon fluxes between land surface and atmosphere, improvement in the understanding of human-environment interactions and influence of changes in one ecosystem on another can be conceived.
